# Healthcare Workers and COVID-19-Related Moral Injury: An Interpersonally-Focused Approach Informed by PTSD

**DOI:** 10.3389/fpsyt.2021.784523

**Published:** 2022-02-14

**Authors:** Andrea M. D'Alessandro, Kimberly Ritchie, Randi E. McCabe, Ruth A. Lanius, Alexandra Heber, Patrick Smith, Ann Malain, Hugo Schielke, Charlene O'Connor, Fardous Hosseiny, Sara Rodrigues, Margaret C. McKinnon

**Affiliations:** ^1^Neuroscience Graduate Program, McMaster University, Hamilton, ON, Canada; ^2^Department of Psychiatry and Behavioral Neurosciences, McMaster University, Hamilton, ON, Canada; ^3^Anxiety Treatment and Research Clinic, St. Joseph's Healthcare Hamilton, Hamilton, ON, Canada; ^4^Department of Psychiatry, University of Western, London, ON, Canada; ^5^Homewood Research Institute, Guelph, ON, Canada; ^6^Imaging Division, Lawson Health Research Institute, London, ON, Canada; ^7^Department of Neuroscience, Western University, London, ON, Canada; ^8^Veterans Affairs Canada, Ottawa, ON, Canada; ^9^Department of Psychiatry, University of Ottawa, Ottawa, ON, Canada; ^10^Centre of Excellence on Post-Traumatic Stress Disorder, Ottawa, ON, Canada; ^11^University of Ottawa Institute of Mental Health Research at the Royal, Ottawa, ON, Canada; ^12^Homewood Health Centre, Guelph, ON, Canada; ^13^Mental Health and Addictions Program, St. Joseph's Healthcare, Hamilton, ON, Canada

**Keywords:** moral injury, post-traumatic stress disorder, healthcare workers, COVID-19, interpersonal factors, social cognition

## Abstract

The COVID-19 pandemic has resulted in a still-unfolding series of novel, potentially traumatic moral and ethical challenges that place many healthcare workers at risk of developing moral injury. Moral injury is a type of psychological response that may arise when one transgresses or witnesses another transgress deeply held moral values, or when one feels that an individual or institution that has a duty to provide care has failed to do so. Despite knowledge of this widespread exposure, to date, empirical data are scarce as to how to prevent and, where necessary, treat COVID-19-related moral injury in healthcare workers. Given the relation between moral injury and post-traumatic stress disorder (PTSD), we point here to social and interpersonal factors as critical moderators of PTSD symptomology and consider how this knowledge may translate to interventions for COVID-19-related moral injury. Specifically, we first review alterations in social cognitive functioning observed among individuals with PTSD that may give rise to interpersonal difficulties. Drawing on Nietlisbach and Maercker's 2009 work on interpersonal factors relevant to survivors of trauma with PTSD, we then review the role of perceived social support, social acknowledgment and social exclusion in relation to potential areas of targeted intervention for COVID-19-related moral injury in healthcare workers. Finally, building on existing literature (e.g., Phoenix Australia—Centre for Posttraumatic Mental Health and the Canadian Centre of Excellence—PTSD, 2020) we conclude with individual and organizational considerations to bolster against the development of moral injury in healthcare workers during the pandemic.

## Introduction

**Figure 1:**
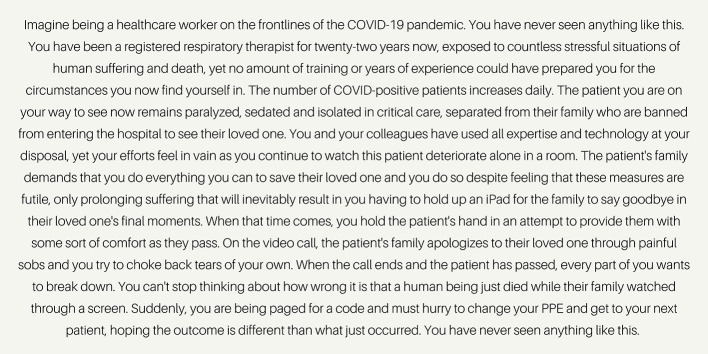
In an ongoing study in our research group, healthcare workers from across Canada have described various events which may be experienced as morally injurious. This vignette provides a summary of the types of events we have heard about from Canadian respiratory therapists early in the Spring of 2021. Participants recounted instances of having to perform care that was perceived to be futile, feeling helpless when caring for critically-ill COVID-positive patients and being at the beside of dying COVID-positive patients in place of their family members, who were prohibited from entering the hospital due to COVID restrictions. Additional stressors compacting upon these potentially morally injurious events included having no time to process events, rushing to change PPE and a high patient caseload.

Healthcare workers around the globe are facing a series of novel, potentially traumatic moral and ethical challenges during the COVID-19 pandemic. In interviews with Canadian healthcare workers that our research group has been conducting throughout 2021, for example, healthcare workers have recounted repeatedly struggling with how *wrong* it feels to helplessly witness the deterioration of human life when caring for critically ill COVID-positive patients (see Figure 1 for a sample vignette of healthcare workers' experiences with moral injury). Exposure to such events has the potential to place healthcare workers at an elevated risk for moral injury. Moral injury is a form of psychological response that may arise when one transgresses, or witnesses another transgress, deeply held moral values, or when one feels that an individual or institution that has a duty to provide care has failed to do so (1, 2). Moral injury is associated with negative mental health outcomes, such as incapacitating feelings of guilt and shame (3) and elevated symptoms of anxiety (4, 5), depression (6–8), post-traumatic stress disorder (PTSD) (4, 5, 9–12) and suicidality (12–14). Despite widespread exposure during the COVID-19 pandemic, limited empirical data renders it unclear at present how best to prevent and, where necessary, treat moral injury in healthcare workers during the pandemic, particularly among those who go on to develop full-blown mental illness as a result of this exposure.

A growing body of literature points to a relation between moral injury and PTSD (reviewed below), suggesting that knowledge in the field of PTSD may translate well to prevention of and intervention for moral injury. Here, an understanding of alterations in social cognitive functioning (e.g., empathy, moral reasoning, theory of mind) associated with PTSD (15) may assist in better elucidating the role of interpersonal factors (e.g., social support, acknowledgment and exclusion) in moderating PTSD symptomology (16). These disruptions are of particular concern where an overwhelming body of evidence points to social support as a consistently strong predictor of who develops PTSD following trauma exposure (17–22), such as the exposures associated with the current pandemic.

Given evidence pointing toward the role of interpersonal factors in moderating symptoms of PTSD, along with the relation between PTSD and moral injury, we suggest here that an interpersonally-focused approach may serve as a useful starting point for prevention, early intervention, and treatment strategies for COVID-19-related moral injury in healthcare workers. Accordingly, the purpose of the present narrative review is to illustrate the potential utility of an interpersonally-focused approach focusing on the role of perceived social support, social acknowledgment and social exclusion as key targets in understanding and mitigating COVID-19-related moral injury in healthcare workers. In this synthesis of the relevant literature, we first review social cognitive impairments previously observed in PTSD that may be associated with interpersonal difficulties. We next consider Nietlisbach and Maercker's (16) landmark review of the role of social support, social acknowledgment and social exclusion in the development and maintenance of PTSD symptoms and consider these factors in relation to moral injury among healthcare workers during the COVID-19 pandemic. Finally, we summarize individual and organizational considerations for bolstering against the development of moral injury among healthcare workers during and after the pandemic.

### Moral Injury in Healthcare Workers

#### Moral Injury and Moral Distress

The potentially traumatic moral and ethical challenges that healthcare workers face in their occupation were first addressed in the moral distress literature. Moral distress has been conceptualized as the psychological distress that arises when a healthcare worker is prevented, by personal or institutional constraints, from doing what they believe to be right (e.g., witnessing the deterioration of patient care due to institutional factors or a lack of communication) (23, 24). Moral distress is associated with poor self-esteem, low job satisfaction, burnout and intention to leave one's position or profession (23–25). Relatedly, moral injury, as reviewed above, has been defined as a type of psychological response to trauma that may arise from exposure to a single or several potentially morally injurious events (PMIEs): rarely occurring, abnormally stressing, high-stakes situations with limited time for decision making (3, 26). Although a concrete definition has yet to be established, Litz et al. (10) contend that PMIEs can take several forms, including acting in ways that contravene moral values (i.e., acts of commission), failing to prevent events that transgress moral values (i.e., acts of omission), or witnessing someone else fail to act in line with moral values (10). Following this definition, PMIEs may be discussed as perpetration- and/or betrayal-based, where an individual holds perceived responsibility for a PMIE (e.g., by acting or failing to act), or has witnessed/been affected by the actions or inactions of others, respectively (11). Morally injurious outcomes will vary on an individual basis according to the codes of moral conduct in one's culture and one's personal values, yet the sequelae of moral injury, as reported in a recent integrative review, often include widespread effects in psychological/behavioral, social, religious/spiritual and biological domains (11). Following Litz and Kerig's heuristic continuum of moral injury (2), moral distress and moral injury may differ in frequency and event magnitude, where moral distress, although harmful, is believed to be less severe in degree of psychological impact when compared to moral injury (2). Due to its origin in the military literature, empirical research on healthcare workers' experiences with moral injury is limited in comparison to research on moral distress among this population. Indeed, in a recent scoping review, Cartolovni et al. (27) identified just seven articles examining moral injury among healthcare workers. Given the unique moral and ethical challenges present in the healthcare arena, exacerbated further by the COVID-19 pandemic, research in this area appears to be accelerating, with some researchers using moral injury and moral distress as interchangeable terms in healthcare during the COVID-19 pandemic (28).

#### COVID-19-Related Moral Injury in Healthcare Workers

Healthcare workers may be at an elevated risk for moral injury during the COVID-19 pandemic as they are more likely to be exposed to PMIEs at this time than, for example, civilians. Possible PMIEs discussed within the healthcare context during COVID-19 include having to take potentially life-saving resources from one patient in an attempt to save another patient's life, exposing individuals to the coronavirus because of failure in the screening process, witnessing healthcare managers poorly ration life-saving resources, or witnessing people living life unbothered outside of the hospital (26, 29). Indeed, preliminary findings from ongoing research in our group have revealed that Canadian respiratory therapists experienced intubating and proning patients over 90 years of age and “holding an infant while he passed away because COVID rules would not allow his mother in the room” as PMIEs. Critically, as the pandemic persists, moral injury and other mental health concerns are expected to remain, if not increase. Hines et al. (30) reported that healthcare workers in the United States showed stable symptoms of moral injury across the first 3 months of the pandemic (i.e., March to July 2020), occurring at levels similar to reports from military veterans upon return from deployment (31). Similarly to military populations, moral injury in healthcare workers during the pandemic has been associated with anxiety, depression, PTSD and suicidal ideation (32). Some caution is warranted in the interpretation of these findings, however, as moral injury is a relatively recent concept in the healthcare context and, to date, the clinical and research communities have not identified a highly-reliable, psychometrically-validated measurement tool for common use in this population.

Notably, as moral injury has been related to psychological/behavioral, social, religious/spiritual and biological harm (11), outcomes of moral injury among healthcare workers performing their duties during the COVID-19 pandemic may also vary across these domains. For example, in the case of a respiratory therapist comforting a dying infant in place of his mother, the respiratory therapist may experience impairing moral emotions of guilt, shame, anger or betrayal. The distress associated with this experience may, in turn, lead the respiratory therapist to withdraw from others, question prior beliefs of the world as a just place or experience a spiritual/existential crisis (11). Exposure to this event may be associated with physical manifestations such as decreased sensitivity to pain or stress-related illnesses, such as arthritis and PTSD, as found in prior research on outcomes of exposure to PMIEs (33, 34). Furthermore, exposure to these types of events may be associated with a sense of being “dirty” or shameful, thus contributing to a sense of being undeserving. Relatedly, such feelings of being “undeserving” have been recounted by healthcare workers in our research group's ongoing interviews during the COVID-19 pandemic where healthcare workers have shared that these feelings have limited their efforts to seek appropriate physical or mental healthcare and/or take breaks and rest periods from the healthcare environment.

### Moral Injury and PTSD

Moral injury and PTSD are currently thought to be associated yet distinct concepts based on symptomology and etiology (9, 11). Both moral injury and PTSD are stressor-linked problems where the outcome is identified after evidence of a prolonged emotional response from exposure to a potentially traumatic or morally injurious event (2). To date, whereas PTSD is considered a mental disorder, moral injury is not (5, 35). There is some evidence to suggest, however, that moral injury and PTSD are associated by symptomology. Based on research with military service members, Litz et al. (10) developed a conceptual model of moral injury that accounts for intersecting symptoms of PTSD and moral injury. In this model, individuals who experience distress over a moral transgression and hold global, internal and stable (i.e., not context dependent, specific to the individual and enduring) attributions about the event are posited to experience enduring shame, guilt and anxiety, which may influence the individual to socially withdraw. With social withdrawal comes failure to encounter experiences with important members of one's community that may otherwise have provided alternative attributions that cultivate self-forgiveness. Here, Litz et al. (10) contend that the path following internal moral conflict, withdrawal and self-condemnation resembles PTSD symptomology. Indeed, chronic intrusions of the morally transgressive event, avoidance behaviors, numbing, self-harming or self-handicapping and demoralization are expected here and, critically, are also classic experiences indicative of PTSD (10, 36).

Conversely, Bryan et al. (12) found evidence of distinct symptom profiles between moral injury and PTSD in a sample of American military personnel. In this study, the PTSD symptom profile included an exaggerated startle reflex, memory loss, flashbacks, nightmares and insomnia, whereas the moral injury symptom profile included guilt, shame, anger, anhedonia and social alienation (12). A distinction between moral injury and PTSD is further supported by etiology, where PTSD has been defined as a response after exposure to direct or indirect life threat or sexual violence (9), but such criteria is not necessary for PMIEs, which are characterized by moral transgressions or betrayal from leadership (10). Furthermore, Currier et al. (33) highlighted how the function of symptoms consistent between PTSD and moral injury may be related to different motivations in some cases. For example, whereas some individuals with PTSD may engage in avoidance behaviors related to fear and safety concerns, some individuals with moral injury may engage in avoidance behaviors motivated by shame and a perception that they may morally contaminate others (33). Although further research on the relationship between moral injury and PTSD is needed, they are currently thought of as associated yet distinct traumatic responses (9, 11).

Given the relation between moral injury and PTSD, it has been suggested that some evidenced-based psychotherapies for PTSD may prove useful in treating moral injury (9, 37). Here, the majority of research and clinical work centred on the treatment of moral injury focuses primarily on military populations (38–41). For example, prolonged exposure therapy and cognitive processing therapy for PTSD have been proposed for treating moral injury in military samples (35, 38, 42). Murray and Ehlers (35), however, recently discussed the use of cognitive therapy for PTSD (CT-PTSD) for moral injury-related PTSD (i.e., PTSD related to traumatic events including a PMIE), providing a case outline and example of the use of CT-PTSD in a healthcare population. As treatment for moral injury continues to be explored, it will be essential that PTSD treatments be strategically adapted to target symptoms specific to moral injury [e.g., dissonance that arises from the discrepancy between moral beliefs and perpetrations/witnessed events, impairing guilt and shame; (10, 12)].

### Social Cognition and PTSD

Alterations in social cognitive functioning have been documented among survivors of trauma who went on to develop a diagnosis of PTSD (15–17, 43–45). Nietlisbach and Maercker (16) previously reviewed evidence for a relation between PTSD and impairments in social cognition and associated interpersonal factors (e.g., social support, acknowledgment and exclusion). Social cognition has been defined as “the ability to use, encode and store information about others that we gain from social interactions” (40). Specifically, social cognition is the coordination of several modes of cognition (e.g., attention, perception, interpretation and processing) in a social context that allows one to perceive and interpret social cues to direct their behavior (41, 46). There are four classic domains of social cognition, namely, theory of mind (ToM), social perception, affective empathy and social behavior (15). ToM refers to the ability to draw on knowledge of how the mind works and of social rules to understand the mental states and beliefs of others (15). ToM can be subdivided into a cognitive component (i.e., what others are thinking) and an affective component (i.e., what others are feeling) (15). Social perception is a domain of social cognition concerned with the ability to recognize and perceive emotional stimuli such as facial expressions, body language or prosody, whereas affective empathy refers to one's emotional response to social situations (15). Finally, social behavior refers to the ways in which an individual conducts themselves in a social context. Alterations in any one of ToM, social perception or affective empathy may lead to deficits in social behavior where an individual displays aggression or socially withdraws (15).

A recently published systematic review of social cognition in PTSD found that social cognition is altered in individuals with PTSD as they display significant impairments in predicting the internal states of others, alterations in perceiving basic emotions and disturbances in empathy (15). In a study investigating emotion recognition and ToM among military police officers exposed to trauma, Mazza et al. (17) found that those with PTSD, in comparison to their counterparts without PTSD, showed deficits in ToM on a task where they were instructed to identify the emotions of a protagonist in a short story. Poljac et al. (44) examined emotion recognition in PTSD patients and controls when viewing video clips of an individual displaying basic emotions. The PTSD group displayed reduced accuracy and sensitivity to facial expressions of fear and sadness in comparison to controls (44). Relatedly, Parlar et al. (47) examined empathic responding among women with PTSD related to childhood trauma. Results of this investigation revealed altered empathic responding in this population, such that women with PTSD showed impairments in identifying the perspective of others when compared to healthy controls (47). Interestingly, however, women with PTSD in this study reported greater levels of personal distress than controls when learning about the negative experiences of others (47). Nazarov et al. (43) examined moral reasoning among women with PTSD related to chronic abuse in childhood and found evidence of altered moral reasoning among these women in comparison to healthy, matched controls. Participants in this study were presented with complex moral situations and were asked to provide a response and justification for their decisions to these dilemmas. The results of this investigation revealed that women with PTSD related to chronic childhood abuse were less likely than controls to approve of utilitarian decisions if the decision involved personally inflicting direct physical harm driven by concern over feelings of guilt and shame associated with these actions (43). Finally, Sherman et al. (45) examined veteran's perceptions of the impact that PTSD had on their parenting, on their children and on the parent-child relationship. More than half of the sample scored above the cut-off on the reactivity subscale of the Parenting Scale, suggestive of alterations in social behavior related to PTSD (45). Cumulatively, the evidence on deficits in key domains of social cognition (i.e., ToM, social perception, affective empathy and social behavior) among individuals with PTSD are related to outcomes of poor quality of life (15). This evidence warrants, in part, the necessity of interpersonally-focused interventions for survivors of trauma with PTSD.

Here, we explore the utility of a social cognitive approach to understanding, mitigating and treating moral injury in healthcare workers during the COVID-19 pandemic. While not intended to be a stand-alone approach to treatment of moral injury in the healthcare population, this perspective may prove useful to the development and implementation of prevention, early intervention and targeted intervention strategies surrounding moral injury in this vital workforce. In a study investigating the relation between moral injury and PTSD among a sample of U.S. National Guard personnel, Bryan et al. (12) noted that a social-cognitive perspective may be a useful approach to understanding moral injury. Social cognitive theory accounts for different types of emotions: natural and manufactured (12, 42). Natural emotions include fear, anxiety and sadness as they are natural responses to direct trauma exposure. By contrast, manufactured emotions are those that arise from an individual's processing and interpretation of events as opposed to the event itself and include feelings of guilt and shame (12, 42). Targeting healthcare workers' interpretation and processing of events during the pandemic, thus, may be one mechanism through which to buffer against the deleterious impacts of guilt and shame related to moral injury. As interpersonal factors such as social support, acknowledgment and inclusion are mediators of PTSD symptom development and maintenance (16), we argue further that an interpersonally-focused approach to moral injury may prove a useful starting point to address COVID-19-related moral injury among healthcare workers.

Importantly, evidence-based treatments for PTSD should always be provided where necessary. The International Society for Traumatic Stress Studies offers guidelines for the prevention and treatment of PTSD, including the use of cognitive behavioral therapy and eye movement desensitization and reprocessing (48). Beyond evidence-based treatments for PTSD that may be adapted for moral injury, a social cognitive approach may be promising, while in need of future study. Notably, this approach relies heavily on a top-down, cognitively oriented approach to the treatment of moral injury. We wish to be clear here that we believe such approaches may, in some cases, require augmentation with more bottom-up therapies that target raw emotion and alterations in somatosensory processes that are also characteristic of PTSD [please see Harricharan et al. (39) for a recent review] (49). An additional caveat to the discussion that follows is that the targeted treatment approach described does not distinguish between dissociative and non-dissociative presentations of PTSD (50–52). Such work, focusing on neuroscientifically-guided approaches to restoration of lower-brain based alterations in emotional processing and somatosensory integration [e.g., deep-brain re-orienting; (53, 54)] as potential augmentative treatments for moral injury are on-going in our research group, as are efforts to develop therapeutic approaches for the treatment of moral injury that distinguish between the dissociative and non-dissociative presentations of PTSD. Lloyd et al. (83) recently demonstrated alterations in top-down control of emotional affect among military and paramilitary personnel with PTSD. Specifically, participants with PTSD described a “nauseating and painful, like an internal gnawing sensation (p. 601)” when recalling morally injurious events, which was thought to be linked to increased activation of the posterior insula and its connections to the viscera. The authors postulated that unpleasant visceral sensations aroused when recalling morally injurious events may, in turn, lead to increased activation of modulating brain areas such as the dorsolateral prefrontal cortex, or the central executive network, in an effort to control excessive bottom-up activity evoked from recalling morally injurious events (83). Critically, over-modulation of excessive bottom-up affect is a pattern of neural activation consistent with dissociation (82, 83). Social support, for example, may then be an important factor in processing morally injurious events as treatments focused on bottom-up affective processes and bodily sensation may encourage pro-social, attachment-based, interpersonal relationships and in turn alleviate interfering symptoms, including heightened arousal and dissociation. Further evidence is required, however, to verify the veracity of this claim and at present, it is imperative that healthcare workers receive access to evidence-based approaches to treat PTSD that, where necessary, may be augmented by evidence-informed approaches for residual symptoms in treatment-refractory cases.

### An Interpersonally-Focused Approach to Moral Injury

With limited empirical data on moral injury in healthcare workers in general and during COVID-19 specifically, we look here to interpersonal factors known to influence the development and maintenance of PTSD symptoms (e.g., social support, acknowledgment and exclusion) as a starting point for potential prevention and treatment strategies. Notably, this interpersonally-focused approach to moral injury is in keeping with evidence from healthcare workers' experiences during the SARS crisis (55). Here, social support and social rejection/isolation were reported to be associated with the psychological impact that the crisis had on healthcare workers (55). Marjanovic et al. (56) found that poor organizational support was associated with avoidance and anger among nurses, a finding similar to that of Tam et al. (57) who reported that poor “team spirit” and administrators not hearing healthcare workers' feedback were associated with poor mental health (55). Similarly, Chen et al. (58) found that nurses who reported greater family support were at a lower risk of mental health problems (55). Koh et al. (59) reported increased work stress and workload among healthcare workers on the frontlines of the SARS crisis in Singapore, with many experiencing social stigmatization and ostracism from family members due to their occupation (55). Drawing on this pre-pandemic evidence in combination with Nietlisbach and Maercker's (16) work on interpersonal factors relevant to survivors of trauma with PTSD, we now provide a review of the role of perceived social support, social acknowledgment and social exclusion in relation to potential areas of targeted intervention for COVID-19-related moral injury in healthcare workers.

### Perceived Social Support

#### Social Support and PTSD

Social support is a psychological construct referring to the emotional and instrumental care provided by those close in one's social circle, such as family or close friends (16). Whereas, seeking social support to deal with traumatic stress is a protective factor against PTSD (18), a perceived lack of social support is strongly associated with increased PTSD symptoms (60). The role of social support in mediating PTSD has been reported among many populations, including war veterans (19), survivors of childhood sexual abuse (20), survivors of violent crime (61) and nurses (21). Cieslak (19) investigated the role of perceived social support and self-efficacy in veterans' adaptations to distress. The results of this investigation revealed that greater received and perceived social support predicted high coping self-efficacy, which in turn predicted lower post-traumatic stress and depression symptom severity (19). Kerasiotis and Motta (21) investigated PTSD symptoms among nurses and found that nurses reported high levels of anxiety but did not reach clinically significant levels of PTSD, depression and dissociation. Here, social support was inferred to help nurses cope with work-related stressors (21). Finally, in a study investigating the specific types of perceived social support that mediated PTSD development in female survivors of childhood sexual abuse, self-esteem support was defined as “others' communications indicating that the abused individual is valued” (20) and was the type of social support that specifically mediated PTSD development in the sample. It is critical to acknowledge that while social support is a strong moderator of PTSD symptom development and maintenance, PTSD characteristically undermines relationships and support networks (62) as discussed above, making social relationships a key target for PTSD intervention. Moreover, PTSD is highly associated with disruptions in childhood attachment (47, 63, 64), rendering it potentially more difficult to form and sustain interpersonal relationships into adulthood.

Social support is thought to influence the cognitive appraisals of traumatic events in survivors of trauma. Specifically, social support may influence how one attributes their role in the traumatic event and their beliefs about the world (65). Cohen and Wills' (66) well-cited stress-buffering model posits that social support is a protective factor against the deleterious effects of trauma exposure as it increases one's perceived ability to cope with trauma and reduces negative appraisals of the traumatic event (22). In a study investigating social constraints, post-traumatic cognitions and PTSD among recent survivors of trauma, those who reported more social constraints (i.e., feeling unsupported, misunderstood or alienated when seeking support) reported more negative post-traumatic cognitions (67). Further, both social constraints and negative post-traumatic cognitions were related to a greater number of PTSD symptoms in this sample (67). These findings are in keeping with decades of research on the role of social support following exposure to trauma where a lack of social support continues to be a strong risk factor for developing PTSD (18, 22, 68). Further, individuals with strong social support are likely to recover faster than those who lack support (68). Indeed, in a recent meta-analytic review, Zalta et al. (22) examined the magnitude of the relation between social support and PTSD symptom severity, reporting a medium effect size across 148 cross-sectional studies and 38 longitudinal studies. The results supported the notion that greater levels of social support and lower levels of negative social reactions are related to lower levels of PTSD symptom severity (22).

Though not fully understood, the relation between social support and resilience documented in neurobiological literature may represent, in part, the mechanism through which social support moderates PTSD symptoms. The noradrenergic and hypothalamic-pituitary-adrenocortical (HPA) systems are implicated in both stress and resilience. Individuals with PTSD display dysregulated noradrenergic systems that fail to terminate the stress response after exposure to stressful stimuli (62). Individuals who report low social support display physiological and neuroendocrine responses, such as increased heart rate and blood pressure, that are indicative of a heightened reactivity to stress (69). Conversely, stress resilience is associated with the ability to keep the HPA and noradrenergic systems stable during exposure to stressful stimuli and terminated when the stimuli are no longer present (69). Social support is thought to influence both biological and environmental susceptibility to stress by acting on the HPA and noradrenergic systems, encouraging resilience (69, 70). As such, on a neurobiological level, social support may be a key factor in mediating the stress response as it promotes resilience.

Relatedly, the neurohormone oxytocin also plays a key role in stress regulation where social contact impacts the HPA axis to release oxytocin and in turn reduce stress (71–77). Indeed, studies using animal models have demonstrated that social contact is associated with oxytocin release (75–77). Furthermore, oxytocin release during or just after exposure to stressful events has been shown to regulate the HPA axis through the corticotropin-releasing factor (72, 78). Oxytocin's affinity for reducing PTSD symptoms may be related, in part, to its critical role in social bonding as oxytocin is known as the neurohormonal substrate of human affiliations, including parental, romantic and filial social bonds (79). For example, oxytocin release is implicated in maternal-infant social bonds such that oxytocin levels early in pregnancy and postpartum were related to maternal bonding behaviors such as positive affect, attachment-related thoughts and frequency of attending to the infant (80). Indeed, in a study investigating the therapeutic potential of intranasal oxytocin administration as a preventative measure for PTSD symptoms, Frijling et al. (81) found that repeated oxytocin administration reduced the development of PTSD symptoms among individuals who were recently trauma exposed. Furthermore, oxytocin has been shown to cause long-term depression of the amygdala, regulating the amygdala's sensitivity to aversive social stimuli (74). As such, the relation between social support and PTSD symptom severity may be explained, in part, due to the release of oxytocin, which is critical for social bonding and regulates the HPA axis along with subcortical structures critical to the stress response [see Lanius et al. (82) for a detailed review of the neurobiology of PTSD, including its dissociative subtype].

#### Healthcare Workers and Social Support During COVID-19

Research on healthcare workers' perceived social support during the pandemic is limited yet provides evidence that social support is critical for mental health and well-being. Indeed, in a systematic review of quantitative studies investigating psychological resilience, coping behaviors and social support in nurses during the pandemic (84), only seven studies explored the relation between social support and mental health outcomes, where greater perceived social support yielded a reduction in burnout (84, 85) and explained variance in psychological distress (84, 86). Using qualitative interviews, Brophy et al. (87) explored Canadian healthcare workers' experiences during the initial months of the pandemic, reporting that a perceived lack of support from employers affected healthcare workers' sense of well-being (e.g., a co-worker being sent to care for a suspected COVID-19 patient without PPE). Similarly, Xiao et al. (88) explored the impact of social support on sleep quality and functioning in a sample of Chinese healthcare workers providing medical care during the pandemic. Social support not only decreased anxiety and stress, but also increased self-efficacy in this sample (88). Finally, Labrague and De los Santos (89) examined the role of resilience, social support and organizational support on COVID-19-related anxiety among nurses working on the frontlines in the Philippines. Here, whereas social support was defined as assistance and protection offered by colleagues, managers, friends and family, organizational support was defined as the degree to which resources, reinforcement, communication and encouragement were offered to an individual by their organization (89). Both social and organizational support significantly predicted COVID-19-related anxiety among this sample, such that greater degrees of these supports were associated with lower degrees of anxiety (89).

In recent surveys of healthcare workers' needs during the pandemic, healthcare workers have highlighted their desire for social support. For example, Shanafelt et al. (90) asked healthcare workers about their main concerns during the pandemic, their needs from leaders and their perspectives on tangible supports. Healthcare workers' requests were summarized as follows: hear me, protect me, prepare me, support me and care for me. Healthcare workers desire clear assurance from their leaders that they, along with their families, will be supported emotionally, physically and socially while working on the frontlines on the pandemic (90). Specifically, healthcare workers shared a desire for their expert perspectives to be included in decision making, for their risk of infection to be mitigated, for appropriate training to treat critically ill patients, for support in dealing with extreme work hours and distress and for practical support such as food and childcare aid should they be infected (90). This call for support was echoed in a recent review by Heber et al. (91) who reported that public safety personnel (PSP) who, like healthcare workers, face novel stressors while working during the pandemic (e.g., greater risk of infection compared to civilians), may benefit from consistent support offered in the form of specialized mental health and preparedness training, frequent and transparent communication, strong leadership and team building, assistance in navigating quarantine and focus on self-care.

Altogether, social support mediates PTSD symptoms in a range of populations, perhaps through influencing cognitive appraisals of events by survivors of trauma (i.e., buffering patterns of common emotional response to trauma, enhancing feelings of connectedness) and likely enhancing resiliency on a neurobiological level [i.e., increasing the availability of neurohormones associated with stress reduction, in turn, regulating the HPA axis and critical subcortical structures associated with trauma and stress; (63–65, 67, 69, 70, 84). Given the relation between moral injury and PTSD symptoms and emerging mental health data suggesting that social support is important for decreasing healthcare workers' anxiety and stress during pandemic situations, targeting perceived social support is one means by which organizations and individuals may mediate the morally injurious outcomes for healthcare workers exposed to PMIEs during the COVID-19 pandemic. This may be accomplished by creating Communities of Practice where healthcare workers may gather in person and/or virtually to discuss shared concerns and coping mechanisms, or by ensuring that healthcare workers have regular breaks to spend with family members or creating “buddy systems.” Similarly, the need for efforts to retain a healthcare workforce that is increasingly considering leaving the profession given experiences throughout the pandemic (92, 93) combined with our own preliminary qualitative findings that healthcare workers demonstrate more concern for team members and family members than themselves, we suggest that efforts to retain healthcare staff and to promote workplace wellness focus, to an extent, on teams and team well-being. Strengthening these connections by highlighting social cohesion with team members may facilitate retention and promote post-traumatic growth. These efforts may also focus on the identity of the healthcare worker as a helping professional and serve as a reminder of why the healthcare worker entered the field in the first place.

### Perceived Social Acknowledgment

#### Social Acknowledgment and PTSD

Social acknowledgment involves receiving appreciation and positive reactions from the wider social environment in recognition of the difficult situation experienced by individuals exposed to trauma (16). The social environment may include colleagues, neighbors, authorities, clergy and the media (94). Social acknowledgment differs from social support as the former measures the degree to which one feels recognized and understood as a survivor of trauma and the latter emphasizes emotional or instrumental care (95).

Poor social acknowledgment (i.e., receiving disapproval and a lack of recognition as a survivor of trauma) is associated with greater severity of PTSD (95). For example, in a study examining PTSD symptom trajectories for Red Cross volunteers in Indonesia who responded to a large earthquake, those in the chronic PTSD trajectory reported lower perceived social acknowledgment compared to those in the resilient trajectory (96). Furthermore, a lack of social acknowledgment predicted increased PTSD symptomology in a longitudinal study on predictors of PTSD recovery in survivors of crime (97). In a study investigating trauma and PTSD symptomatology in German developmental aid workers, some participants reported that experiencing general disapproval from others was associated with more severe intrusive thoughts about the traumatic event and increased hyperarousal (94). Finally, a study on PTSD symptom severity, disclosure attitudes and social acknowledgment among Chinese and German survivors of crime found that, although PTSD symptom severity differed cross-culturally, disclosure attitudes and social acknowledgment predicted PTSD severity in both groups where a reluctance to disclose and a perceived lack of social acknowledgment predicted greater PTSD severity (97).

One mechanism through which social acknowledgment may mediate PTSD symptom development and maintenance is similar to that of social support, namely, post-traumatic cognitions. Post-traumatic cognitions are recognized as strong predictors of PTSD (97). Whereas, a strong sense of social acknowledgment may help survivors of trauma affirm positive cognitions that a traumatic experience has damaged, poor social acknowledgment may foster negative self- and other-focused cognitions (95). Indeed, Mueller et al. (97) found that perceived social acknowledgment was negatively associated with post-traumatic cognitions in a sample of 86 survivors of crime.

Here, it is critical to note evidence of altered patterns of response to emotions among individuals with PTSD that may pose as a barrier to the reception of social acknowledgment. For example, Nazarov et al. (98) found evidence for altered comprehension of affective prosody among women with PTSD related to chronic child abuse. Women in this study were asked to recognize angry, fearful, sad and happy emotions on a computer-based task and also asked to identify if the emotions portrayed in consecutive excerpts were the same or different. Nazarov et al. (98) found that women with PTSD took longer to identify all emotions except for those portraying anger in comparison to healthy controls. Interestingly, women with PTSD who experienced dissociative symptoms were more likely to be less accurate in discriminating between consecutive emotional presentations (98). Further, greater severity of childhood trauma was related to poorer accuracy in discrimination as well as slower recognition of emotions (98). Consistent with the altered patterns of emotional response demonstrated by Nazarov et al. (98), alterations in ToM, emotional recognition, empathic reasoning, moral reasoning and social behavior have been demonstrated among individuals with PTSD, as described above (15, 17, 43–45). As such, altered patterns of emotional response in PTSD must be considered when evaluating interventions targeting social acknowledgment.

#### Healthcare Workers and Social Acknowledgment During COVID-19

Research on healthcare workers' perceived social acknowledgment during the pandemic is limited, with the majority of work instead focused heavily on social support from familiar others, such as co-workers. A small number of studies, however, have explored healthcare workers perceptions surrounding a lack of recognition for their efforts during the COVID-19 pandemic. For example, some healthcare workers have described feeling abandoned by political leaders and have attributed inadequate staffing and PPE during the pandemic to political leaders' poor response to their needs during the crisis (26, 99). Relatedly, in a recent systematic review and meta-synthesis on frontline healthcare workers' experiences during pandemics and epidemics (e.g., COVID-19, SARS, MERS, Ebola), Billings et al. (100) reported that workers felt abandoned and betrayed by organizations when they did not received promised financial renumeration for their service and sacrifice on the frontlines. Kröger (26) stated further that healthcare workers may perceive an inconsistent world outside of the hospital when witnessing people carelessly ignoring safety measures. Despite window decorations in homes and businesses thanking healthcare workers for their service, healthcare workers may perceive the non-chalance of people in their communities as a lack of recognition for their sacrifice on the frontlines. Finally, Cai et al. (99) investigated the psychological impact and coping strategies of healthcare workers in China and found that recognition from management and the government was associated with psychological benefit. Preliminary findings from our research groups' ongoing interviews with healthcare workers throughout 2021 corroborate the perceived lack of recognition discussed in the literature where nurses discussed feeling underappreciated at the beginning of the pandemic when financial renumeration for their service was smaller in proportion to that offered to some first responders. Unsurprisingly, we also heard from some healthcare workers that they felt abandoned and described a lack of recognition from the community when protests against masks and vaccines were held outside the hospital.

As social acknowledgment plays a role in the development and maintenance of PTSD symptoms in various trauma-exposed groups, it is imperative that healthcare workers perceive that they are recognized and understood as survivors of trauma during the COVID-19 pandemic. This perception may influence positive post-traumatic cognitions that may ameliorate moral injury as a response to COVID-19-related-PMIEs. Indeed, in outlining how healthcare workers desire strong leadership to support them during the pandemic, Shanafelt et al. (90) note the critical role of leadership asking healthcare workers about their concerns and acknowledging their requests. Despite potentially being constrained from providing answers, leadership must demonstrate that healthcare workers' service is acknowledged and appreciated (90). This call for strong leadership was echoed by Jetly et al. (1) who discussed key qualities of effective leadership in the military that may prove useful for leaders attending to COVID-19-related moral injury in healthcare workers. Jetly et al. (1) propose that effective leadership listens to the concerns of subordinates, are positive yet not overly optimistic, respect their subordinates' values, recognize that the risk of COVID-19 infection is not evenly distributed as frontline workers are the most vulnerable at this time, care about those whom they lead and accept the blame for team failures while attributing success to their team members.

### Perceived Social Exclusion

#### Social Exclusion and PTSD

Social exclusion is an act of ostracism related to stigmatization where one is rejected and isolated from a group (16). Humans have an inherent need for relationships with others that is disturbed in situations of social exclusion (101). Social exclusion may lead to feelings of isolation as if one were no longer viewed as a part of society (16). When ostracized, survivors of trauma may perceive themselves as less-human and believe that they are perceived to be less-human by the perpetrator of their ostracism (101). Social exclusion or ostracism can lead to physiological changes, such as cardiovascular issues or increased cortisol levels and psychological issues, including negative emotions of anger, sadness and shame (102, 103).

Individuals suffering from PTSD and other mental illnesses are often victims of social exclusion in the form of stereotyping and stigmatization (104). For example, among East-African conflict survivors, stigmatization was associated with an increased likelihood of PTSD after exposure to trauma (105). Wesselmann et al. (103) investigated the relation between perceived ostracism (i.e., “being ignored and excluded”) and post-traumatic stress among military veterans and found that perceived ostracism was related to post-traumatic stress symptoms, anxiety and psychological distress. Further, perceived ostracism explained variance in post-traumatic stress symptoms apart from theoretically relevant variables (i.e., deployment stress and social support) (103). In a study investigating the effects of social exclusion between individuals with PTSD and control participants, those with PTSD reported greater perceived social exclusion than control participants in an experimental manipulation of inclusion and exclusion (106), perhaps underscoring evidence of impaired social cognition among individuals with PTSD. Finally, among female adolescents exposed to war-related trauma, those who experienced sexual violence reported increased stigmatization (i.e., feeling treated worse than others, being insulted, rejected and excluded from family or community) (107). Further, stigmatization explained symptoms of depression and post-traumatic stress more so than did the direct impact of sexual violence in this sample (107).

#### Healthcare Workers and Social Exclusion During COVID-19

Healthcare workers may perceive themselves to be socially excluded during the pandemic. In acknowledgment of the stigmatization that healthcare workers may experience by their communities during disease outbreaks, Taylor et al. (108) conducted a North American study evaluating non-healthcare workers' attitudes toward healthcare workers serving during COVID-19 and found that more than one quarter of respondents believed that healthcare workers should be subject to severe restrictions, including isolation from communities and families. Further, one-third of the respondents reported avoiding healthcare workers due to fear of infection (108). Indeed, Kröger (26) highlighted how healthcare workers have been prohibited from certain public spaces including bringing their children to school and Shimizu and Lin (109) recounted instances of defamation against healthcare workers in Japan during the pandemic. Individuals affected by COVID-19 in Japan have experienced societal rejection, discrimination and stigmatization and since healthcare workers are at a high risk of infection due to contact with COVID-19 patients, they are more likely to suffer from social exclusion (110). Reports indicate that Japanese healthcare workers have been victims of discrimination and abuse outside of work as the public treats them as “germs” (109). Healthcare workers in Japan have also been denied access to public transportation and their families have fallen victim to discrimination as well (109). In Canada, a national news organization reported on two nurses' experiences of facing criticism for crossing the border to provide care at a hospital in Detroit. The nurses described being blamed for bringing COVID cases into their city as they worked to be a part of the solution by caring for COVID-19 patients (111). Furthermore, Dye et al. (112) conducted a survey of healthcare workers' experiences of COVID-19-related bullying and stigma with participants from across 173 countries, reporting that healthcare workers were experiencing social exclusion during the pandemic, especially in communities affected by the intersection of racism, violence and police involvement (112).

In sum, social exclusion violates the inherent human need for relationships, consequently generating physiological and psychological disturbances. The effects of social exclusion in the form of stigmatization have been documented among various populations such as war veterans (103), survivors of sexual violence (107) and survivors of conflict (105). Our research group is currently preparing a scoping review on healthcare workers' and public safety personnel's exposure to PMIEs and distressing experiences during the pandemic. Here, we identified a need for further investigation of the verbal and physical abuse that healthcare workers have experienced in order to understand the context and severity of such exposures globally. As social exclusion is implicated in the development and maintenance of PTSD symptoms, targeting healthcare workers' perceptions of social exclusion may be an effective strategy to prevent and treat COVID-19-related moral injury.

## Discussion

In Litz et al.'s (10) conceptual model of moral injury, it is clear that an individual's social world is expected to mediate their response to PMIEs. Indeed, this model postulates that the degree of dissonance an individual initially experiences post-PMIE will be moderated by others' reactions. In addition, stable, internal and global attributions may be countered when an individual has others in their environment to offer interpretations of events that lead to self-forgiveness rather than self-condemnation (10). Targeting perceived social support, acknowledgment and inclusion may be an opportunity for PMIE-exposed healthcare workers to reconcile dissonant cognitions of their beliefs about the world and their experiences. Here, this targeted approach may aid the individual in processing intense moral emotions and deter them from social withdrawal, which may otherwise manifest into symptoms of moral injury such as self-condemnation, overwhelming shame, guilt, anger, anhedonia and/or PTSD symptoms. For example, social exclusion may play a critical role in how healthcare workers evaluate themselves after experiencing perpetration-based PMIEs during the COVID-19 pandemic. In interviews our research group has conducted with healthcare workers throughout 2021, healthcare workers have described excessive guilt when not able to attend to patients quickly enough due to changing out of and into PPE. If an individual in this situation is subsequently ostracized from colleagues or receives condemning information from close individuals in their life regarding their actions, these reactions may strengthen beliefs such as “I am a bad person” and “I am responsible for the patient's death”, contributing to the sequalae of moral injury postulated by Litz et al. (10).

Relatedly, social support and acknowledgment may not only be factors that mediate morally injurious outcomes among those who are exposed to PMIEs, but may also inherently constitute PMIEs for some healthcare workers during the pandemic. For example, in a recent review of frontline healthcare workers' perspectives on working during pandemics and epidemics (e.g., COVID-19, SARS, Ebola), Billings et al. (100) discussed how healthcare workers valued support from their organizations and perceived the organization to have “an institutional duty to provide staff with sufficient protection to work safely” (pg. 10). Healthcare workers may feel betrayed by their organization when they are sent to care for COVID-19 patients without appropriate PPE (87) or when they feel unheard by leaders (91) who establish procedures and policy with which workers do not agree. Finally, actions or inactions that communicate to healthcare workers that their efforts on the frontlines are not recognized or appreciated [e.g., lack of inclusion in financial compensation; (100)] may constitute betrayal-based PMIEs for some healthcare workers. Thus, healthcare organizations must carefully consider the ways in which their operations may betray their employees in addition to recognizing that, as a trusted authority to their healthcare workers, they have the ability and responsibility to support workers and mitigate the effects of moral injury through means of social support and acknowledgment.

An important consideration in translating research on interpersonal factors in PTSD to moral injury, however, is that moral injury by definition may involve witnessing morally transgressive acts and/or an individual enacting the moral harm themselves. Thus, the utility of addressing social support, acknowledgment and exclusion in moral injury may need to be strategically implemented to accommodate for both personally transgressed and witnessed PMIEs, which has not been explored in the PTSD literature. This tailored approach will mimic research that has adapted traditional PTSD treatments to better suit the experience of those with moral injury (11, 35). With this consideration, it is necessary for future studies to continue to investigate the boundaries between moral injury and PTSD as well as the ways in which a social cognitive model can inform treatment interventions.

An interpersonally-focused approach to understanding and mitigating moral injury in healthcare workers during COVID-19 is consistent with a recent call for moral injury treatments to move beyond psychotherapy and include an affirmative community effort (11). This approach must include both individual and organization considerations that take into account the unqiue indiviudal in their specific social environement. Indeed, Billings et al. (100) highlighted the importance of the social environment when reviewing the experiences of frontline healthcare workers affected by stigma and discrimination in their communities. Here, healthcare workers reported feeling supported by their orgnizations at times (e.g., when communication was clear, when buddy systems were established between expereinced and new staff) and unsupported at other times (e.g., when staff safety was not a clear priority, when they felt coerced to caring for infected paitents). Below, we synthesize and disucss ciritical considerations for bolstering against the deliterious imapcts of moral injury in healthcare workers at both the indiviudal and organizaiton level.

### Individual Considerations

In line with the recommendations made in the recent Moral Injury Guide prepared by the Centre of Excellence on PTSD (113), we offer the following individual level considerations to buffer against the deleterious impacts of moral injury for healthcare workers during the COVID-19 pandemic.

At the individual level, it is important to consider risk and resilience factors that may affect one's likelihood of developing moral injury after exposure to a PMIE. Williamson et al. (114) highlighted risk factors for moral injury in the military, which may be pertinent to healthcare workers during the pandemic, including loss of life to a vulnerable person (e.g., elderly, children), perception of leaders not taking responsibility for events or being unsupportive, staff feeling unprepared for the emotional or psychological impacts of decisions, concurrent exposure to other traumatic events and a lack of social support after exposure to a PMIE. Furthermore, our research group has shown that emotional abuse during childhood (115, 116) and emotional regulation (116) are critical factors related to moral injury. Battaglia et al. (115) examined the relation between childhood abuse and moral injury among members of the Canadian Armed Forces (CAF) and found that emotional abuse in childhood may increase the likelihood of moral injury among adults in the CAF (115). Further, Roth and colleagues (116) demonstrated that exposure to adverse childhood experiences was associated with both moral injury and trauma-related symptoms among PSP, but emotion regulation skills buffered moral injury (116). Thus, whereas healthcare workers who are survivors of childhood trauma may be at an elevated risk for moral injury, those with strong emotion regulation skills may have the necessary resilience to buffer against the development of moral injury.

Healthcare workers who identify with a minority group (e.g., gender, sexual orientation, or racial/ethnic group) may also be at an elevated risk for moral injury during the pandemic due to the compacting nature of minority stress (i.e., stress associated with discrimination or marginalization) with COVID-19-related stressors. The Centre of Excellence on PTSD's moral injury guide (113) highlighted that racialized individuals are at a higher risk of COVID-19 exposure, infection and severe outcomes due to racial disparities in income and poverty. Further, racialized healthcare workers may deal with stressors related to systemic racism in addition to the stressors associated with providing care during COVID-19. For example, racialized healthcare workers may experience racism from patients and colleagues, may witness colleagues make racist comments about patients, may feel compelled to protect racialized patients from racism, may face skepticism about their training and competence, may experience belittlement of their speech, appearance, religion or cultural practices and may be called upon to educate others about systemic racism (113). As a result, racialized healthcare workers may be at an increased risk of experiencing distress and moral injury during the pandemic in comparison to their non-racialized counterparts. It is vital to acknowledge the unique stressors that healthcare workers who identify with minority groups may face in relation to discrimination and marginalization and consider these impacts when developing interventions for COVID-19-related moral injury.

Based on Heber at al.'s (91) recommendations for PSP to support themselves during COVID-19, healthcare workers may benefit from taking time to practice self-care in the form of healthy coping and seeking connections and help. For example, frontline workers should prioritize sleep, nutrition, hydration and exercise, stress management, relaxation, maintaining/establishing routines, connecting with friends, family and co-workers and seeking informal and formal supports as needed (91). These individual recommendations were echoed in the Centre of Excellence on PTSD's guide to moral injury (113), which additionally recommends that healthcare workers gain education about moral stressors, moral injury, relaxation therapy and mindfulness or meditation to reduce stress.

### Organizational Considerations

Cohen and Wills' (66) stress-buffering model contends that a specific match between trauma and the subsequent social support is necessary for social support to reduce the harmful psychological responses associated with trauma exposure. Thus, in the context of COVID-19, healthcare organizations must play a critical role in supporting healthcare workers to buffer against moral injury as their workers look to leadership for guidance in these unprecedent times. In line with the recommendations made in the recent Moral Injury Guide prepared by the Centre of Excellence on PTSD (113), we offer the following organizational level considerations for leaders to consider in an effort to buffer against the deleterious impacts of PMIE exposure and moral injury as an outcome for healthcare workers during the COVID-19 pandemic. Recommendations for organizations to support healthcare workers include: offering clear, positive yet realistic information; establishing peer support networks among healthcare workers where team cohesion is emphasized; time and space to rest and discuss experiences; encouraging self-forgiveness and re-integration of moral transgressions into one's moral code; honest discussions about the moral requirements of working during a pandemic; leaders taking responsibility to ensure that staff are prepared for the emotional consequences of their work and are aware of vicarious traumatization and relevant coping strategies; peer support teams to provide psychological first aid; encouraging staff to utilize employee assistance programs, chaplaincy, or other levels of support; intentionally expressing gratitude to frontline workers; offering accessible professional resources; rotating staff between high and low stress roles; optimizing the work environment for appropriate breaks; educating healthcare workers on PMIEs and the possible morally injurious outcomes that may be experienced after exposure (e.g., overwhelming moral emotions of anger, guilt, shame betrayal; self-condemnation, anhedonia, social withdrawal), encouraging self-assessments for risks of moral injury and formal screening for PTSD, as well as receiving evidenced-based treatment, if required (113, 117–121). Critically, some healthcare workers may show signs of distress, yet deny or lack insight into these difficulties and refuse help (122). Healthcare workers involved in previous pandemics and epidemics did not fully voice their needs or seek supports until after the peak of the crisis (100). As such, it is essential that orgnaizations continiously offer and encourage staff to prioritize their mental health during and beyond the COVID-19 pandemic, even in the absence of explicit requests for this support.

### Limitations

While a thorough search of the literature was conducted in preparing this review, it must be acknowledged that the present review is limited by its narrative structure, which inherently is unsystematic and is consequently associated with bias. The purpose of this review was to synthesize literature on different topics (e.g., social cognition, PTSD, moral injury) to present the notion of an interpersonally-focused approach to COVID-19-related moral injury in healthcare workers. Though it has limitations, a narrative review was the most appropriate type of review for this work (123). The present review is additionally limited by the paucity of empirical research surrounding moral injury in healthcare workers and a lack of consensus on moral injury as a clinical phenomenon. As moral injury has historically been situated in the military context, there remains a need for research on the types of events that may be experienced by healthcare workers as PMIEs, as well as the outcomes associated with these exposures, to better characterize healthcare workers' experiences and arrive at a definition for moral injury in this population. Future research should consider a systematic review of the literature on moral injury in healthcare workers as data becomes available to reduce bias and synthesize knowledge on moral injury in this population.

## Conclusion

In summary, many healthcare workers have faced morally and ethically challenging situations throughout the COVID-19 pandemic that place them at an elevated risk for moral injury. The relation between moral injury and PTSD, although not fully understood, suggests that the two are related yet distinct responses to trauma. Alterations in social cognitive functioning that may contribute to interpersonal difficulties in PTSD render interpersonal factors such as social support, acknowledgment and exclusion important in moderating PTSD symptomology. Existing research on the interpersonal factors that moderate PTSD symptom development and maintenance, then, may prove to be a useful starting point for preventing and, where necessary, treating moral injury in healthcare workers during the COVID-19 pandemic. We urge researchers and clinicians to consider carefully a social cognitive and interpersonal lens in relation to ongoing research on moral injury in healthcare workers during the COVID-19 pandemic and beyond. Healthcare organizations should follow the available recommendations to support healthcare workers at this time to bolster against moral injury.

## Author Contributions

AD'A: conceptualization, writing (original draft), review, and editing. KR and RM: conceptualization and review and editing. RL, AH, PS, AM, HS, CO'C, FH, and SR: review and editing. MM and RL: conceptualization, writing, review and editing, and funding acquisition. All authors contributed to the article and approved the submitted version.

## Funding

This work was supported by research grants from the Veteran's Affairs Canada supported Centre of Excellence on PTSD, Defense Canada, and the Canadian Institute of Health Research (grant number: MVP-171647) to MM and RL, along with a generous donation from Homewood Health Incorporated to Homewood Research Institute. MM was supported by the Homewood Chair in Mental Health and Trauma at McMaster University. RL was supported by the Harris-Woodman Chair in Psyche and Trauma at Western University of Canada.

## Conflict of Interest

The authors declare that the research was conducted in the absence of any commercial or financial relationships that could be construed as a potential conflict of interest.

## Publisher's Note

All claims expressed in this article are solely those of the authors and do not necessarily represent those of their affiliated organizations, or those of the publisher, the editors and the reviewers. Any product that may be evaluated in this article, or claim that may be made by its manufacturer, is not guaranteed or endorsed by the publisher.
